# Retraction Note: lncRNA TINCR sponges miR-214-5p to upregulate ROCK1 in hepatocellular carcinoma

**DOI:** 10.1186/s12881-020-01178-9

**Published:** 2021-03-12

**Authors:** Min Hu, Yaowu Han, Ying Zhang, Yuanfeng Zhou, Lin Ye

**Affiliations:** 1 Department of Pathology, Anhui University of Chinese Medicine, 1 Qianjiang Road, Hefei, 230012 Anhui Province China; 2 Graduate School, Anhui University of Chinese Medicine, Hefei, 230038 Anhui China; 3 Department of Psychology, Anhui University of Chinese Medicine, Hefei, 230012 Anhui China

**Retraction to: BMC Med Genet 21, 2 (2020)**

**https://doi.org/10.1186/s12881-019-0940-6**

The Editor has retracted this article [1] due to lack of evidence that the study has received ethics approval.

After publication it has come to the Editor’s attention that in the body of the article the authors state that their study was approved by the Ethics Committee of Anhui University of Chinese Medicine. However, in the Ethics declarations section, the authors state that their study was approved by the Ethics Committee of the Maternity and Child Care Center of Liuzhou.

The authors have not provided documentation of approval from an ethics committee for this study.

Author Min Hu stated on behalf of all co-authors that they agree to this retraction.

## References

[1] Hu M, Han Y, Zhang Y (2020). lncRNA TINCR sponges miR-214-5p to upregulate ROCK1 in hepatocellular carcinoma. BMC Med Genet.

